# Effect of Water–Ethanol Extraction as Pre-Treatment on the Adsorption Properties of *Aloe vera* Waste

**DOI:** 10.3390/ma15165566

**Published:** 2022-08-13

**Authors:** Leone Mazzeo, Irene Bavasso, Melissa Spallieri, Maria Paola Bracciale, Vincenzo Piemonte, Luca Di Palma

**Affiliations:** 1 Department of Chemical Engineering Materials & Environment, Sapienza University of Rome, Via Eudossiana, 18, 00184 Rome, Italy; 2 Department of Engineering, University Campus Biomedico of Rome, Via Alvaro del Portillo, 21, 00128 Rome, Italy

**Keywords:** *Aloe vera*, adsorption, extraction, methylene blue, mass transfer

## Abstract

The adsorption properties of *Aloe vera* (*Aloe barbadensis Miller*) for the uptake of Methylene Blue (MB) from water were investigated after pre-treating the material with water–ethanol solutions at different ethanol concentrations: 0% *v*/*v* (AV0), 25% *v*/*v* (AV25), and 50% *v*/*v* (AV50). The pre-treated materials were characterized as follows: the pHZC was evaluated to be 6, 5.7, and 7.2 for AV0, AV25, and AV50, respectively; from BET-BJH analysis the mesoporous nature of the material and an increase from 108.2 (AV0) to 331.7 (AV50) m^2^/kg of its solid surface area was observed; TG analysis revealed a significat increase in volatile compounds from the untreated (5.4%) to the treated materials (8.9%, 10.3%, and 11.3% for AV0, AV25, and AV50, respectively). Adsorption batch tests were then performed to investigate the equilibrium, the kinetics, and the thermodynamics of the process. Results suggested that the Langmuir model was in agreement with the experimental results, and values for q_max_ of 199 mg/g, 311 mg/g, and 346 mg/g were calculated for AV0, AV25, and AV50, respectively. The kinetic results were used to develop a mathematical model to estimate the effective diffusion coefficient for each type of Aloe adopted. Effective diffusion coefficients of 5.43·10^−7^ cm^2^/min, 3.89·10^−7^ cm^2^/min, and 5.78·10^−7^ cm^2^/min were calculated for AV0, AV25, and AV50, respectively. It was found that pre-treatment, on the one hand, enhances the adsorption capacity of the material and on the other, reduces its affinity toward MB uptake.

## 1. Introduction

The removal of pollutants from wastewater is one of the most significant environmental challenges of the century. The lack of available water sources and tightening of regulatory standards of permissible component levels make wastewater treatment essential and mandatory. Pollutants are characterized by a very wide range of toxicological profiles and physio-chemical properties. Dyes can perturb the biological activities of algae and plants by hindering light penetration in water. Moreover, they can develop allergies, dermatitis, and even cancers, through contact with the gastrointestinal tract, skin, and lungs [1]. Methylene Blue (MB) is a cationic dye commonly used for dying cotton, wood, and silk [2]. It can cause difficulty breathing in cases of inhalation and eye burning in cases of eye contact, while it is responsible for nausea, vomiting, profuse sweating, mental confusion, and methemoglobinemia when swallowed [3].

The most used processes for the removal of dyes from wastewater are sedimentation (using a coagulating/flocculating agent) and filtration (microfiltration, ultrafiltration, nanofiltration, and reverse osmosis). However, such techniques are far from approaching the minimal liquid discharge (MLD) or zero liquid discharge (ZLD) scenario [4] since they generate a concentrated sludge/brine, which still poses arising environmental concerns [5,6]. Other possible solutions to dye pollution remediation are biodegradation, oxidation, and advanced oxidation. The main drawback of such processes is that they require severe control of the operating conditions (such as pH), and they deal with the risk of missing the total mineralization/degradation of the pollutants [7].

Hence, most wastewater treatment technologies present several issues and have high operational and maintenance costs (i.e., replacement costs) [8]. Adsorption is a valid alternative for its ease of operation and simplicity of design and also because it avoids the formation of toxic sludge.

Although activated carbons are recognized worldwide as the most used and performant adsorbents for wastewater treatment [9], their high activation cost [7] lead us to search for new low-cost, environmentally friendly adsorbents. In particular, agro-industrial wastes seem to be promising alternatives [10] since they abate the cost related to the synthesis of commercial adsorbents, promoting, at the same time, a circular economy.

*Aloe vera* (*Aloe barbadensis Miller*) is a perennial, drought-resistant succulent herb known as the healing plant or the silent healer because of its wound and burn healing properties. The *Aloe* leaf consists of three layers, i.e., gel (inner layer), latex (middle layer), and rind (outer layer) [11]. The gel is made of 99% water, amino acids, lipids, sterols, vitamins, and polysaccharides (such as glucomannans), which are associated with most of the health benefits of *Aloe* [12]. For this reason, industrial interest is mainly focused on collecting the *Aloe vera* gel, rejecting both latex and rind, producing 400 kg of solid waste per month in the local agroindustry of Yucatàn and Mexico [13]. However, gel residues, together with other valuable components, are also present in other layers, such as anthraquinones contained in the latex [11]. Several studies were conducted for the extraction of high-value molecules from *Aloe vera*. For example, Acemannan was extracted from both gel and *Aloe* skin with water or water–ethanol solutions as explained in the review of Liu et al. [14] or anthraquinones were extracted from *Aloe* peel powder in a 60% ethanol–water solution by Tan et al. [15].

*Aloe vera* wastes were also used for water treatment purposes, mainly for the removal of dyes and heavy metals [16]. Giannakoudakis et al. [17] pointed out different classes of pre-treatment steps before the utilization of *Aloe* as a bio-adsorbent: no treatment; air-dried treatment at T < 110 °C; chemical treatment with strong acids; thermal treatment at 300 °C < T < 700 °C from which activated carbons or ash are obtained depending on the absence or the presence of oxygen respectively. However, all the aforementioned pre-treatments do not focus on the recovery of valuable compounds that are still present in the Aloe waste matrix (i.e., anthraquinones), which could be recovered, promoting economic advantages. For this reason, solvent extraction has been selected as the best pre-treatment option, which could simultaneously achieve increased adsorption properties of the material and benefits from an economic point of view.

To the best of our knowledge, there are no studies that utilize solvent extraction on *Aloe vera* as a pre-treatment for the preparation of new bio-adsorbent material. Although the aim of this work was far from optimizing the particular extraction step adopted, it was hoped to provide the first analysis of its influence on the adsorption properties of waste material. Thus, the purpose of this work is to couple an extraction (as a pre-treatment) step using solvent water or a solution of ethanol–water (adopted for extraction on *Aloe* as previously explained) followed by batch adsorption of Methylene Blue and to investigate how the adsorption properties of *Aloe vera* change depending on the solvent used. 

In the work of Hanafiah et al. [18], *Aloe vera* rind powder washed with water was tested as an adsorbent material of Methylene Blue; however, several points of interest were not studied: no information was provided regarding the amount of substances released during the washing step with water; the equilibrium data are focused in a concentration range which is below 25 mg/L, and possible information about the behavior of the system at higher concentration was lost; no physical model was developed for the analysis of the adsorption kinetics. 

In this work, the equivalent Gallic Acid concentration (GA) was quantified after the pre-treatment step, and experimental data of adsorption equilibrium with Methylene Blue are reported for the material treated with different extracting solvents. Moreover, experimental data are also reported regarding the batch adsorption kinetics of Methylene Blue from the aqueous phase. Such data were analyzed by a mathematical model to estimate the Methylene Blue diffusion coefficient inside the bio-adsorbent and to compare the results related to the different pre-treatment steps.

## 2. Materials and Methods

### 2.1. Chemicals

Methylene Blue (C_16_H_18_ClN_3_S; MB), pure ethanol (C_2_H_5_OH), hydrochloric acid (HCl), sodium hydroxide (NaOH), sulfuric acid (H_2_SO_4_), sodium carbonate (Na_2_CO_3_), and Folin Ciocalteou reagent (C_10_H_5_NaO_5_S) were purchased from Sigma Aldrich (St. Louis, MO, USA) and used without any further purification.

### 2.2. Bio-Adsorbent Preparation

The outer layer (rind) of *Aloe vera* (*Aloe barbadensis Miller*) leaves was obtained from residues of a cosmetic industry located in the Lazio region, Italy. The solid was firstly dried at 60 °C for 24 h in order to remove the moisture initially present. Then, it was drilled and sieved in order to collect particles in a size range of 0.5–1 mm. Three different aqueous solutions were tested for the pre-treatment: water, water–ethanol (25% *v*/*v*), and water–ethanol (50% *v*/*v*). The acronyms AV0, AV25, and AV50 were adopted to identify the *Aloe vera* treated with water, with the water–ethanol (25% *v*/*v*), and with the water–ethanol (50% *v*/*v*) solution, respectively. The pre-treatment step was carried out at room temperature in 500 mL magnetically stirred flasks with a solid concentration of 20 mL/g (in the range adopted by Gironi et al. [19] and Fanali et al. [20]) for 2 h. The total amount of organic compounds in the aqueous solution was evaluated by Total Organic Carbon (TOC) measurement (TOC-L Analyzer- Shimadzu), while the equivalent Gallic Acid (GA) concentration was measured by means of the Folin Ciocalteou reagent (using gallic acid as standard). The latter (0.144 mL) was mixed together with 5 mL of HCl (0.1 M), 4.656 mL of Na_2_CO_3_ (10% *w*/*w*), and 0.2 mL of the sample. The solution was continuously reacted in the dark for 1h before spectrophotometric analysis using a PG Instrument (Leicestershire, United Kingdom) T80+ UV/Vis spectrophotometer (with glass cells of 1 cm path length) at a wavelength of 700 nm. 

### 2.3. Bio-Adsorbent Characterization

The zero-charge point pH of the pre-treated materials (pH_ZC_) was determined according to the pH drift method [16]. An amount of 0.04 g of Aloe were added to 10 mL of ultra-pure water. Before the introduction of the solid, the pH of the solution was adjusted from 2 to 12 by the addition of H_2_SO_4_ (0.1 M) and NaOH (0.1 M). The pH of the solution was then measured (Crison GLP 421) after 24 h, which was estimated to be the time required for the system to reach the equilibrium condition. Analysis of the adsorbent’s surface area was performed by N_2_ adsorption isotherms acquired at −196 °C using a Micromeritics Triflex analyzer (Micromeritics Instrument Corp., Norcross, GA, USA) in the p/p^0^ range from 0.01 to 0.99. Isotherm analyses were carried out using 3Flex Version 4.05 software. Samples were previously outgassed at 100 °C for 3 h. The Brunauer–Emmett–Teller (BET) and Barrett–Joyner–Halenda (BJH) equations were used to determine the specific surface area (SSA), pore volume, and average pore diameter, respectively. A thermal-gravimetric analyzer (TGA-SDTQ600, TA Instruments, New Castle, DE, USA) was employed to investigate the degradation profile of the prepared samples over a temperature range of 30–700 °C. The dried *Aloe vera* samples were ground into a very fine powder using a non-metallic electric grinder, and approximately 10 mg were placed onto a platinum crucible and heated at 10 °C/min under 100 mL/min N_2_ flow. 

### 2.4. Batch Tests

To evaluate the affinity between the solid adsorbent and the model compound (MB) to be removed, batch tests were performed. In all the tests, the concentration of MB was determined by an Infinite M200 PRO Tecan microplate spectrophotometer (Tecan Trading AG, Männedorf, Switzerland) at a wavelength of 664 nm.

#### 2.4.1. Equilibrium Tests

All equilibrium experiments were carried out in magnetically stirred beakers of 100 mL contacting 50 mL of MB solution of known concentration in the range of 8.5 mg/L–212.5 mg/L. Each test lasted at least 24 h since preliminary tests showed that it was enough time for the system to reach equilibrium. The stirring speed remained constant at 220 rpm while the solid dosage was fixed to 0.2 g/L unless in the case of 212.5 mg/L MB starting solution in which it was set to 0.4 g/L. The temperature was fixed to 20 ± 0.2 °C. Each test was performed for AV0, AV25, and AV50 alone or in duplicate. Only for AV0 was the equilibrium evaluated at 10 ± 0.2 °C and 40 ± 0.2 °C in order to understand the nature of the adsorption mechanism. The solid concentration at equilibrium ($q_{e})$ was calculated from experimental data as follows:

(1) $$q_{e}=\frac{V}{M_{S}}\left(C_{in}-C_{e}\right)$$

where V (mL) is the liquid volume, $M_{S}$ (g) is the mass of the adsorbent material, $C_{in}$ (mg/L) is the initial concentration of MB, and $C_{e}$ (mg/L) is the MB concentration at equilibrium.

#### 2.4.2. Kinetic Tests

All kinetic experiments were carried out in magnetically stirred beakers of 250 mL contacting 100 mL of MB solution at a starting concentration of 8.5 mg/L. The stirring speed was kept constant at 220 rpm while the temperature was set at 20 ± 0.2 °C, verifying that such condition was satisfied before the beginning of the tests. The solid to liquid ratio was fixed to 0.2 g/L. Samples were taken at definite time intervals by removing 200 µL from the liquid. Since the number of samples collected was less than 10, the error made on the final volume was <2%. Each test was performed for AV0, AV25, and AV50 alone, or in duplicate. A mathematical model (deeply discussed in the following section) was adopted to fit the experimental data by means of the process simulator gProms (Process System Enterprises, London, UK) and to evaluate the controlling resistance of the system.

## 3. Results and Discussion

### 3.1. Bio-Adsorbent Pre-Treatment

**Figure 1:**
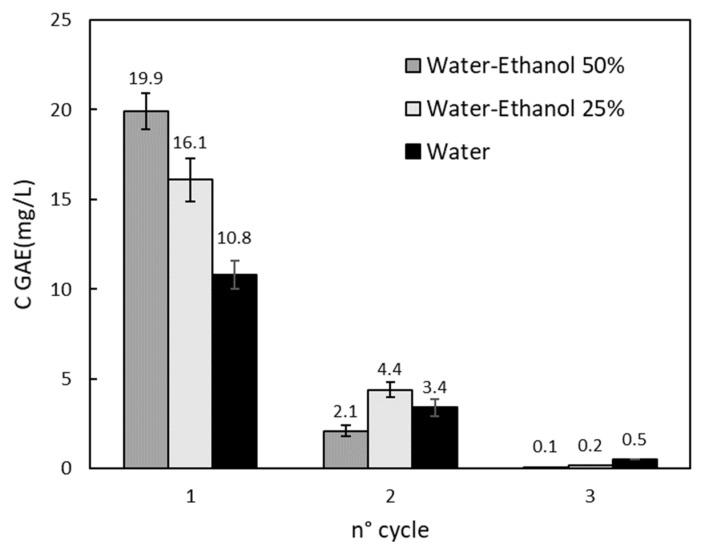
Equivalent Gallic Acid concentration (C GAE) along three pre-treatment cycles using water, water–ethanol (25% *v*/*v*), and water–ethanol (50% *v*/*v*) solutions as solvents.

Solid preparation with only drying and milling steps is not sufficient to obtain a clean material to be adopted for adsorption tests because of the interference of released compounds during the tests on MB spectrophotometer detection. For this reason, different pre-treatments (as washing cycles) were adopted, and the effect of the washing solution composition was investigated. For this purpose, water and a water–ethanol solution (25% *v*/*v* and 50% *v*/*v* of ethanol) were adopted. The pre-treatment procedure was repeated, replacing fresh solvent with the same amount of solid until the concentration of polyphenols in the liquid phase reached the detectable limit of the spectrophotometer. The results of the pre-treatment are reported in Figure 1, where it is possible to show that three cycles were enough to reach such a threshold. The total amount of polyphenolic compounds (TPC) was evaluated to be 0.4 mg/g of equivalent GA per gram of Aloe. A similar result was observed by Di Scala et al. [21] for a pre-treatment conducted at 0.1 Mpa. In Figure 1, it is also noticeable that the water–ethanol solutions have a higher extraction potential in agreement with the higher solubility of polyphenolic compounds in such mixtures with respect to water alone [22,23]. Furthermore, Figure 1 highlight the decrease of the GA concentration along the pre-treatment cycles. To evaluate the concentration of the total organic compounds that can be removed from AV solid (not only limited to polyphenolic compounds), the TOC of the final solution was measured: this was conducted for the only water test, and a value of 79.4 mg/L (10% of which were polyphenols) was observed as a sum of the three washing cycles. This result suggests that many other components are extracted and, in an attempt to adopt such pre-treatment as possible extraction process of added value compounds, optimization of the operative parameters (temperature, solid/liquid ratio) of the process and purification of the extracts are required. The aim of this work was to study the adsorption properties of AV waste, even in the case of residue as a product of the extraction process. 

**Figure 2:**
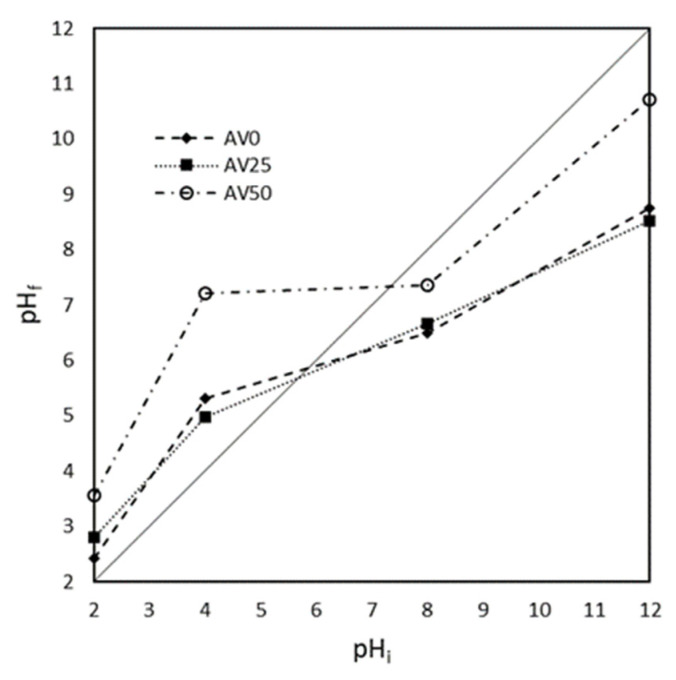
Zero-point charge (pHZC) determination for AV0, AV25 and AV50. The zero-point charge is represented by the intersection of the characteristic lines for all the samples with the bisector of the graph.

The pH_ZC_ was evaluated graphically and reported in Figure 2: values of 6, 5.7, and 7.2 for AV0, AV25, and AV50, respectively, were determined. As reported in Figure 2, AV0 and AV25 displayed similar behaviors, while AV50 showed a higher affinity for protonation. These results suggest that the pre-treatment could induce some changes in terms of protonating/deprotonating functional groups on the solid surface when solvent at a higher percentage than 25% *v*/*v* with respect to the water adopted. 

**Figure 3:**
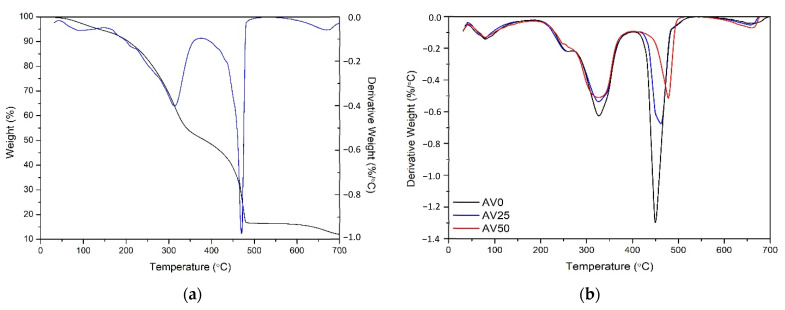
(**a**) TG-DTG curves of as-received *Aloe vera*; (**b**) DTG curves of AV0, AV25, and AV50.

Figure 3a show the TG curve of as-received *Aloe vera* waste. The first weight loss region in this curve can be seen between room temperature and 150 °C, which is due to the removal of adsorbed/bound water and other trapped volatile compounds, which constitute approximately 5.4 wt% of the total weight. The second weight loss region is observed at 150–380 °C, and this corresponds to the decomposition of hemicellulose and pectin (43.6 wt%). Beyond 380 °C, weight loss is very fast due to the presence of cellulose (34.5 wt%). The last slow weight loss occurring between 540 °C and 750 °C is due to the presence of lignin, which accounts for approximately 4.8 wt% of the total weight. The amount of solid residue remaining at 700 °C was about 11.6 wt% [18]. In Figure 3b, the DTG curves of AV0, AV25, and AV50 are reported, and a decrease in thermal stability of the pre-treated materials is observed with the increase of ethanol percentage adopted in the pre-treatment solutions. No significant modifications, such as the hemicellulose, cellulose, and lignin amount, were detected, and values of 51.83%, 30.26%, and 3.40% were determined, respectively. The difference with respect to the untreated one is related to the reduction of solid residue in the analyzed samples from 11.60% ± 0.84% to 4.33% ± 0.61%, contributing to an increase in the percentage of these species with respect to the untreated AV. A significant increase in volatile compounds was measured from the untreated (5.4%) to the treated materials (8.9%, 10.3%, and 11.3% for AV0, AV25, and AV50, respectively), probably due to the strong absorption of ethanol on the active sites of lignocellulosic material. With the increase of ethanol content (higher than 25% *v*/*v*) in the pre-treatment solution, the modification in terms of pH_ZC_ must be associated with a possible protonation/deprotonation ethanol mechanism [24].

### 3.2. Equilibrium

**Figure 4:**
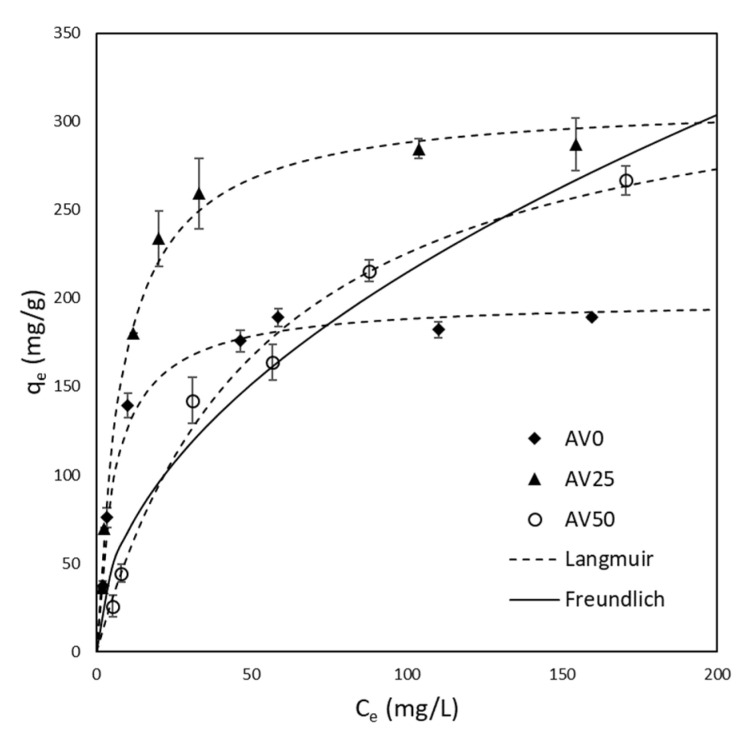
Isotherms at 20 °C for AV0, AV25, and AV50 fitted with Langmuir model. Only for AV50 was the fitting provided with the Freundlich model.

The equilibrium experimental data for the bio-sorbents AV0, AV25, and AV50 are reported in Figure 4. It is immediately noticeable that the isotherms for AV0 and AV25 showed typical saturation behavior. Hence, the Langmuir model (Equation (2)) was chosen to analyze the experimental data of these two isotherms and only for completeness was a comparison made also using the Freundlich model (selected for its wide usage in literature). Different considerations should be made for the AV50 isotherm: in the range of concentration considered, it was not possible to affirm that the curve reaches saturation. For this reason, both Langmuir and Freundlich (Equation (3)) models must be considered for data description. The Langmuir equation is provided below in Equation (2).

(2) $$q_{e}=q_{max}\frac{bC_{e}}{1+bC_{e}},$$

where b (L/mg) is the equilibrium constant of the Langmuir model correlated to the affinity of binding sites and $q_{max}$ (mg/g) is the maximum adsorption capacity. The Freundlich equation is also reported in Equation (3).

(3) $$q_{e}=k_{F}C_{e}^{1/n_{F}},$$

where $k_{F}$ ((mg/g)(L/mg)^(1/n^_F_^)^) is the distribution coefficient and $1/n_{F}$ is a correction factor. According to such considerations, the experimental data were fitted by means of non-linear regression using the least square method. The Langmuir and the Freundlich parameters $q_{max}$, b, $k_{F}$, and $1/n_{F}$ were estimated for AV0, AV25, and AV50 and were reported in Table 1 together with their corresponding root mean square error (RMSE). According to such results, it was observed that the Langmuir isotherm provides a better representation of the system for all the samples under analysis. For this reason, the Langmuir model was selected as a representative also for the thermodynamic analysis of the adsorption mechanism discussed later in this section. In Figure 4, it was chosen to only plot the curve obtained for AV50 from the regression with the Freundlich model since, for AV0 and AV25, the graphical results were unsatisfactory. Moreover, in Table 2, the adsorption capacities of other bio-sorbents regarding the uptake of MB were collected and compared with the ones obtained in this study. From Table 2, it is possible to observe that all the pre-treated Aloe Vera wastes considered in this work showed good performances for the adsorption of MB, particularly sample AV50.

Table 1 show that both *Aloe* AV50 and *Aloe* AV25 have a higher adsorption capacity than AV0 of 74% and 56.5%, respectively, toward the uptake of Methylene Blue from water. Moreover, the value of $q_{max}$ of AV50 with respect to the one of AV25 is 11.13% higher. It is possible to ascribe this enhancement in terms of *q_max_* to the increase of exposed surface area, as demonstrated by the results of the BET analysis shown in Table 3. (N2 adsorption isotherms for each pre-treated material (AV0, AV25 and AV50) and the related pore volume distributions see the Supplementary material Figures S1 and S2). This result, linked to the pre-treatment operated with water–ethanol solution, is not related to the solubilization of polyphenolic compounds (that were completely removed as discussed in the previous section) but to other soluble molecules present in the AV rind matrix such as tannins [37] whose solubilization increases with the percentage of ethanol in water [23].

**Figure 5:**
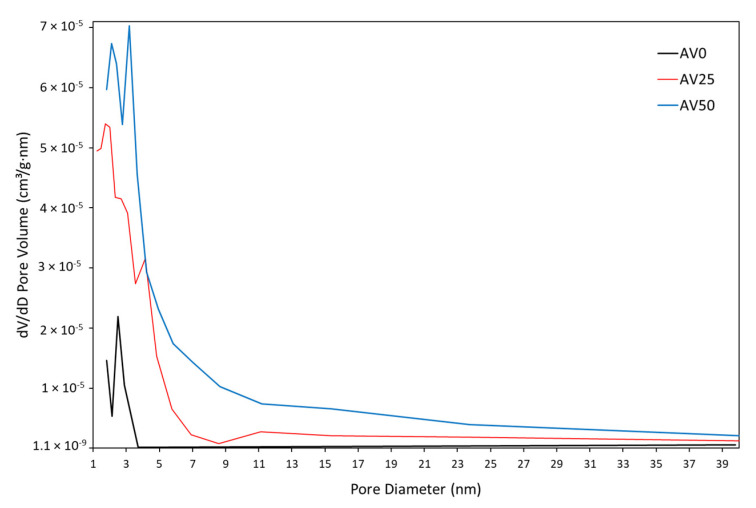
Relation between pore volume and diameter for AV0, AV25, and AV50.

Using the International Union of Pure and Applied Chemistry (IUPAC) definition for microporous (less than 2 nm in diameter) and mesoporous (less than 50 nm in diameter) materials, the pore diameter patterns reported in Figure 5 and Table 3 showed that all samples are mainly mesoporous materials. This result is in agreement with the study of El-Azazy et al. [38].

As reported in Table 1, a decrease in the Langmuir equilibrium constant b was observed. In fact, the reduction of the parameter was 29.4% (AV25) and 89.4%(AV50) with respect to AV0. This result suggests that pre-treatment in the presence of ethanol, on the one hand, enhances the solubility of species and, as a consequence, increases the sites available for adsorption, but, on the other hand, reduces the affinity with MB molecules, significantly in the case of AV50 in line with the observation on pH_ZC_.

Considering a batch system having an initial concentration $C_{in}$ of MB in the liquid solution, with only knowledge of thermodynamic information, it is possible to calculate the concentration of the solution and hence the percentage of MB removed (R%) at equilibrium by solving Equations (1) and (2) together as follows:

(4) $$R=1-\frac{C_{e}}{C_{in}}=1-\frac{1}{2}\left[-\left(\frac{1}{bC_{in}}+\frac{M_{S}q_{max}}{V_{L}}-1\right)+\sqrt[{2}]{{\left(\frac{1}{bC_{in}}+\frac{M_{S}q_{max}}{V_{L}}-1\right)}^{2}+\frac{4}{bC_{in}}}\right].$$

**Figure 6:**
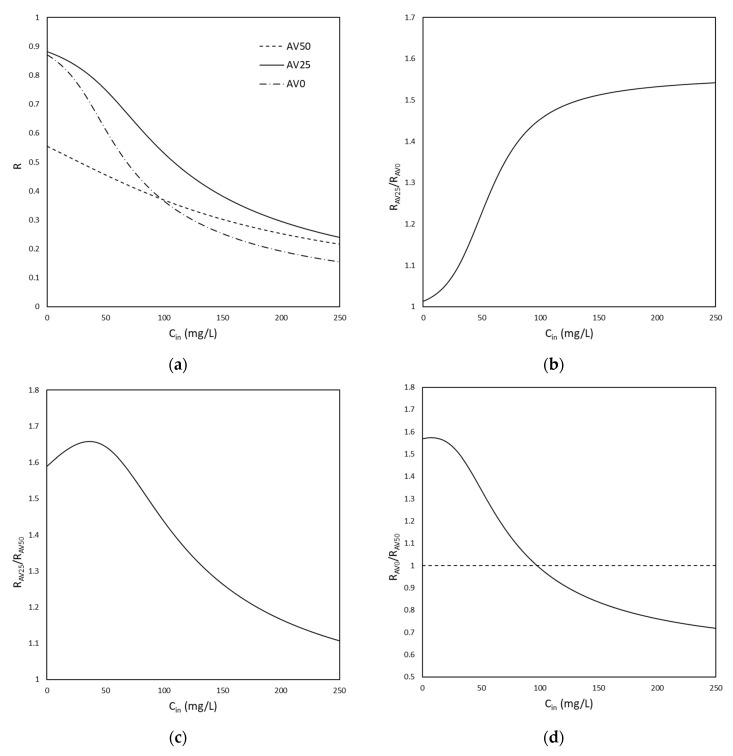
(**a**) Plot of the removal percentage at equilibrium of MB (R) vs. the initial concentration for AV0, AV25, and AV50; (**b**) plot of the ratio $R_{AV25}$ /$R_{AV0}$ versus the initial concentration; (**c**) plot of the ratio $R_{AV25}$ /$R_{AV50}$ versus the initial concentration; (**d**) plot of the ratio $R_{AV0}$ /$R_{AV50}$ versus the initial concentration.

Figure 6a report the R calculated for AV0, AV25, and AV50 for different initial concentrations of MB. As it is possible to notice in Figure 6a, the value of R for AV25 is always bigger than the one for AV0, as expected due to the higher adsorption capacity of AV25. For this reason, as shown in Figure 6b, the ratio between the R referred to as AV25 ($R_{AV25}$) and the one referred to as AV0 ($R_{AV0}$) is always >1, independent of the variation of $C_{in}$. The same scenario occurs in Figure 6c as a consequence that for all the range of $C_{in}$ explored, AV25 removes a higher amount of MB with respect to AV50. Moreover, in Figure 6c, the presence of a maximum is also noticeable, which indicates the initial concentration of MB (which was calculated to be 50 mg/L) for which the bio-adsorbent AV25 shows the best performance with respect to AV50.

Figure 6a also shows that $R_{AV50}$ and $R_{AV0}$ intersect each other. This means that depending on the starting concentration of MB, AV50 may perform better than AV0 and vice versa. In fact, in this case (Figure 6d), the ratio between $R_{AV0}$ and $R_{AV50}$ is not always >1: $C_{in}<100\;mg/L$, $\frac{R_{AV0}}{R_{AV50}}>1,$ and AV0 removes more MB than AV50; $C_{in}>100\;mg/L$, $\frac{R_{AV0}}{R_{AV50}}<1,$ and AV50 removes more MB than AV0. The range of initial concentration explored is limited to the one analyzed experimentally.

**Figure 7:**
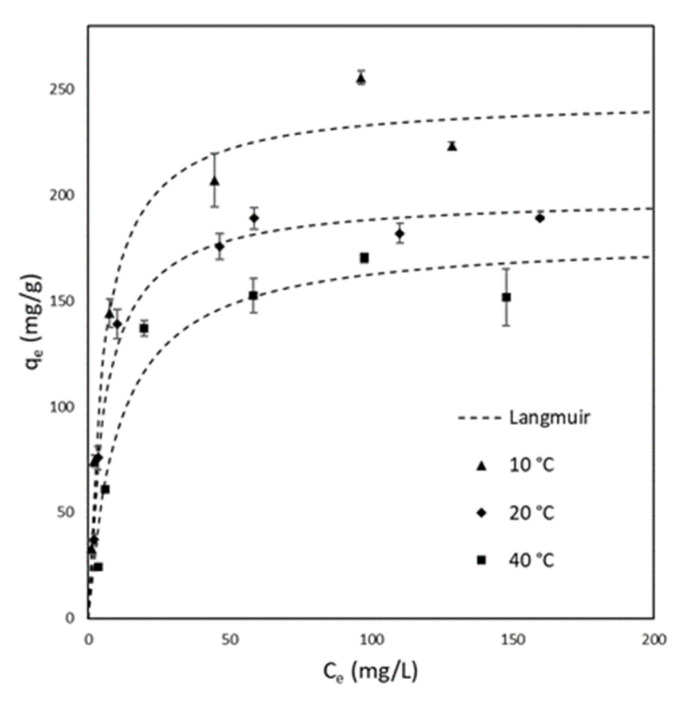
Isotherms of AV0 at 10 °C, 20 °C, and 40 °C fitted by the Langmuir model.

AV0 was chosen as a representative sample to understand the nature of the adsorption mechanism since it has a higher affinity toward MB adsorption with respect to the other samples considered. Moreover, to evaluate if, in the case of AV0, it is possible to promote the adsorption mechanism, the effect of temperature was considered. Figure 7 report the equilibrium data for AV0 at different operative temperatures (10 °C, 20 °C, 40 °C). In addition, for this case, the data were analyzed by means of the Langmuir isotherm. The values of $q_{max}$ and b were calculated for each temperature and are provided in Table 4. It can be immediately observed that there is a decrease in both $q_{max}$ and b with temperature.

**Figure 8:**
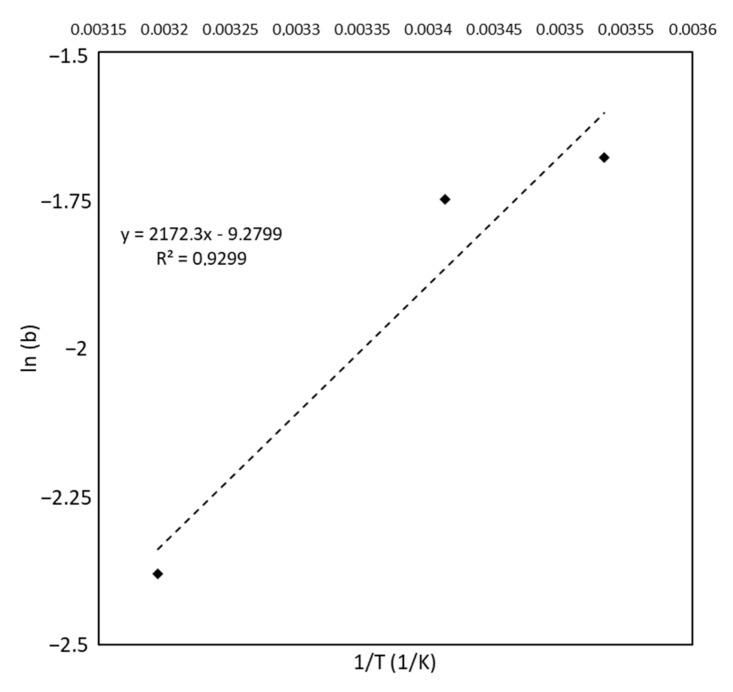
Natural logarithm of the Langmuir constant *b* versus the reciprocal of temperature. The dotted line represents the result of the linear regression, for which the equation is reported in the graph together with the value of R^2^.

An increase in the$\;q_{max}$ value was calculated, but such results are lower with respect to those related to AV25 and AV50 at 20 °C. On the contrary, *b*, even to a small extent, increased. According to the Langmuir theory of adsorption, b represents the equilibrium constant of a reversible reaction between the target compound and the active sites. For this reason, the equilibrium constant *b* can be related to the enthalpy variation of the process by means of the Van ’t Hoff equation that leads to the following relation:

(5) $$ln\left[b\left(T\right)\right]=C-\frac{\Delta h}{R}\frac{1}{T},$$

where *C* is a constant that arises from the integration of the Van ’t Hoff equation, R (J/mol K) is the universal gas constant, T (K) is the temperature, and Δh (J/mol) is the specific enthalpy variation assumed to be constant with temperature. Equation (5) predicts a linear relationship between the natural logarithm of the equilibrium constant against the reciprocal temperature. As shown in Figure 8, the experimental data behave as suggested in Equation (5). By means of linear regression, the value of Δh was calculated to be −4.3 kcal/mol, which is included in the typical range associated with the physical adsorption of −1 to −10 kcal/mol [39]. Moreover, the variation in enthalpy is negative, revealing the exothermic nature of MB adsorption on *Aloe,* as expected from the decrease of b with the increase of the temperature (see Table 4).

As previously observed, the adsorption capacity $q_{max}$ decreases with temperature. Despite the fact that the Langmuir isotherm describes the equilibrium conditions of the system, the Langmuir theory does not admit a change in $q_{max}$ with temperature. In fact, Langmuir assumes a monolayer coverage of the solid surface with a constant amount of active sites available, which is not related to the system conditions but only to the morphology of the adsorbent material. Even if the variation of $q_{max}$ with temperature is quite common in adsorption of dyes (and in particular of Methylene Blue), this aspect is often neglected (for example see [36,40,41,42]). In this work, the aim was to highlight this topic underlining the lack of a rigorous theoretical isotherms suitable to describe the adsorption of electrolytic non-volatile compounds such as dyes. However, Polanyi’s theory associates physical adsorption with a phenomenon of solid precipitation [43]. According to this point of view, the “apparent” increase of adsorption capacity at lower temperatures may be due to a decrease in the salt solubility in water. 

### 3.3. Kinetic Tests

The dynamic model used to describe the adsorption of MB on the types of *Aloe* considered (AV0, AV25, and AV50) was based on the following assumptions:

- isothermal conditions;
- the batch system was perfectly mixed;
- solid particles were assumed to be spherical with a constant radius;
- equilibrium conditions were assumed at the solid–liquid interface.

Moreover, it was considered that pore diffusion was the main intra-particle mass transfer mechanism. The validity of this choice was confirmed by the mesoporous structure of *Aloe* (as observed in the previous section).

According to such a hypothesis, the mass balance of Methylene Blue in the liquid solution is:

(6) $$\frac{dC_{MB}^{L}}{dt}=-k_{L}a\left(C_{MB}^{L}-C_{MB}^{L,i}\right)$$

where $C_{MB}^{L}$ (mg/L) and $C_{MB}^{L,i}$ (mg/L) represent the concentration of Methylene Blue in the liquid bulk and at the solid–liquid interface, respectively, $k_{L}$ (cm/s) is the mass transfer coefficient across the liquid film, and a (cm^2^/mL) is the specific surface area of the solid particles per volume of liquid.

The microscopic material balance in a solid particle is:

(7) $$\frac{\partial q}{\partial t}=D_{eff,MB}\left[\frac{2}{r}\frac{\partial q}{\partial r}+\frac{\partial^{2}q}{\partial r^{2}}\right]$$

where q (mg/g) is the solid concentration of MB and $D_{eff,MB}$ (cm^2^/s) is the MB effective diffusion coefficient. The mathematical connection between Equations (6) and (7) was realized by introducing the following macroscopic balance:

(8) $$\frac{V_{L}}{M_{S}}\left(C_{in}-C_{MB}^{L}\right)=\frac{1}{\frac{4}{3}\pi R^{3}}\int_{0}^{R}q\left(r\right)4\pi r^{2}dr$$

where R (cm) is the radius of a solid sphere. The initial and boundary conditions used were as follows:

(9) $$IC:\;\;t=0\;\;\forall r\;\;q=0;\;C_{MB}^{L}=C_{in},\;\;$$

(10) $$BC:\;\;t>0\;\;r=0\;\;\frac{\partial q}{\partial r}=0;\;\;r=R\;\;q=q_{max}\frac{bC_{MB}^{L,i}}{1+bC_{MB}^{L,i}}\approx q_{max}bC_{MB}^{L,i}.\;$$

An opportune initial concentration (8.5 mg/L) was selected to operate in the linear region of the isotherm (see Figure 4) in order to avoid the dependence of $D_{eff,MB}$ from concentration [44]. For this reason, it was possible to simplify the isotherm expression in Equation (10), neglecting the possible errors derived from such an assumption. Taking into account the latter consideration, the effective diffusion coefficient can be written as follows:

(11) $$D_{eff,MB}=\frac{D_{p}}{\varepsilon +\rho_{s}\;q_{max}b}$$

Where $D_{p}$ (cm^2^/s) is the MB pore diffusion coefficient, ε is the porosity of the solid, and $\rho_{s}$ (g/L) is the solid adsorbent density.

Once the mathematical model was defined, the values of $k_{L}a$ and $D_{eff,MB}$ were then estimated by fitting the kinetic experimental data using the maximum likelihood method

Commonly, the fitting of such a model is focused on finding $k_{L}$ alone [45], which it is possible to estimate by knowing the density of the solid ($a=\frac{3}{R}\frac{M_{S}}{V_{L}}\frac{1}{\rho_{S}}$ where $\rho_{S}$ is the solid density). Since it may be difficult to determine the solid density for waste-derived adsorbent materials, it was decided to fit the entire term $k_{L}a$ to obtain sufficient information on the liquid film transfer resistance. The value of the particle diameter used in the model was 0.075 cm (the average of the particle size range). 

**Figure 9:**
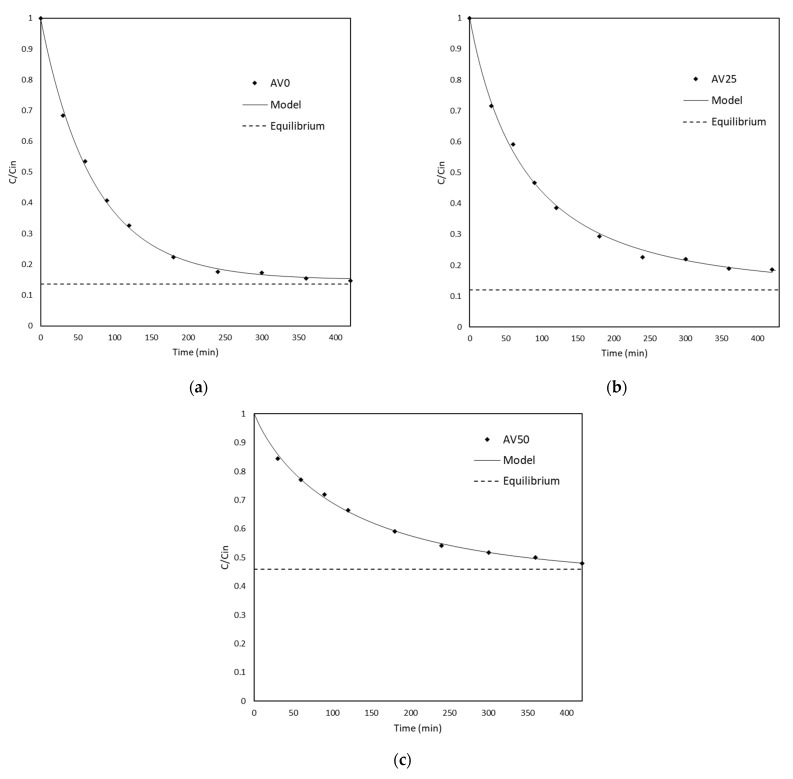
Experimental kinetic data for (**a**) AV0, (**b**) AV25, and (**c**) AV50 reported as the ratio between the concentration of Methylene Blue in water and its initial concentration (C/Cin) versus time. The solid line represents the model prediction, while the dotted line is the expected equilibrium threshold.

Figure 9 report the kinetic data for AV0, AV25, and AV50 in terms of the variation of the ratio between the concentration of MB in water and its initial concentration. It was decided not to report the error bars in the graph because of the very small experimental error observed. As it is possible to notice in Figure 9, the theoretical model represents the experimental behavior of the system well. Furthermore, the $\chi^{2}$ test was performed to verify the goodness of fit for each estimation imposing a significance level of α = 0.05 (data not shown).

In order to detect how far the system was from equilibrium at the end of the kinetic test, the ratio between the concentration at equilibrium with respect to the initial concentration of the system was calculated by Equation (4) and plotted in the graph. 

The values of $k_{L}a$ and $D_{eff,\;MB}$ derived from fitting are reported in Table 5. As expected, the $k_{L}a$ calculated for AV0, AV25, and AV50 are of the same order of magnitude since the fluid dynamic conditions set in each test were the same. In particular, the $k_{L}a$ estimated for AV0 is 7.6% smaller than the one obtained for AV25 and 62.5% higher than the one obtained for AV50. This result could be attributed to some oscillation of the average particle size, which in the model was chosen to be 0.75 mm, but that can be included in the range of 0.5–1 mm (as explained in the Materials and methods section) or by the lack of setting perfectly the same agitation speed for each test.

As shown in Table 5, the effective diffusion coefficient $D_{eff,MB}$ follows the order of AV50 > AV0 > AV25. In contrast with the common feeling that the effective diffusion coefficient must increase with the material porosity, which is true only for the pore diffusion coefficient $D_{p}$ (for information about the pore volume of the materials see Table 3), $D_{eff,MB}$ was higher for AV0 than for AV25. This behavior can be easily explained by means of Equation (11) in which the relation between $D_{eff,MB}$ and ε, $q_{max}b$ is provided. It was discovered that in this case, the effective diffusion coefficient follows the same order of $1/q_{max}b$ for each material which is again AV50 > AV0 > AV25. In other words, it was found that the equilibrium terms rather than the porosity play a major role in the determination of the effective diffusion coefficient. 

The values calculated in this work for the effective diffusivities of Methylene Blue are comparable with the ones found in the literature. For example, Sáenz-Alanís et al. [46] estimated a diffusivity of 1.7 · 10^−7^ cm^2^/min for the adsorption kinetics of MB on commercial bituminous granular activated carbon (GAC) and diffusivity of 2.8 · 10^−7^ cm^2^/min for the adsorption kinetics of MB on heat-treated activated carbon (HGAC).

The evaluation of the limiting resistance of the process was performed by calculating the dimensionless Biot number. The latter is defined as the ratio between the characteristic time of diffusion in the solid ($\tau_{D}$) with respect to the characteristic time of mass transfer ($\tau_{t}$) from the liquid to the solid phase. By imposing the continuity of the mass flux at the solid boundary [47], the following expression was obtained:

(12) $$Bi=\frac{\tau_{D}}{\tau_{t}}=\frac{R^{2}}{D_{eff,\;MB}}k_{L}a\frac{V_{L}}{M_{S}}\frac{C_{in}}{q_{e}\left(C_{in}\right)}$$

where $q_{e}\left(C_{in}\right)$ (mg/g) is the solid concentration in equilibrium with the initial concentration of MB ($C_{in}$) in the liquid bulk. As shown in Table 5, the values of the Biot number for AV0, AV25, and AV50 are all Bi>1 and hence $\tau_{D}>\tau_{t}$. This result indicates that the controlling resistance of the adsorption process is the intra-particle one. However, in this case, the value of Bi is still not big enough (Bi∈[1;100] [48]) to neglect the external mass transfer resistance for the description of the adsorption kinetics.

## 4. Conclusions

This paper investigated the effects that a water–ethanol pre-treatment (not optimized) induces on the adsorption performances of an agro-waste material. In particular, *Aloe vera* rind was treated with water alone (AV0), with water–ethanol 25% *v*/*v* (AV25), or with a water–ethanol 50% *v*/*v* (AV50) solution. It was observed that with the increase of the ethanol concentration in the pre-treatment solution, an increase in the adsorption capacity of AV50 and AV25 of about 74% and 56.5% higher than the one of AV0, respectively, was calculated and supported by BET-BJH analysis. On the other hand, the affinity of *Aloe vera* with respect to the adsorption of MB (represented by the Langmuir constant) dramatically decreased when ethanol was used following the order AV0 > AV25 > AV50. This phenomenon was attributed to the possible adsorption of ethanol on the material surface during pre-treatment. As a matter of fact, TGA analysis revealed a major presence of volatile compounds in the Aloe matrix treated with a higher percentage of ethanol. By means of thermodynamic analysis, the physical nature of the adsorption mechanism was also confirmed. The implementation of a mathematical model was used to calculate the effective diffusion coefficient for AV0, AV25, and AV50, and it was found that the affinity toward MB plays a major role in its final value. A dimensionless analysis method from which the number of Biot was calculated was also developed. It was concluded that both intra-particle and film transfer mechanisms are important in the adsorption kinetics in the operating conditions adopted. 

The results obtained increase the knowledge of the effects that a possible extraction step may induce on the adsorption performances of agro-alimentary waste material. Moreover, it opens a new perspective for the two-fold valorization of *Aloe* waste material. Further studies are necessary to analyze both the extraction step and the adsorption step in a continuous operation mode.

## Figures and Tables

**Table 1:** Langmuir and Freundlich isotherm parameters at 20 °C for AV0, AV25, and AV50.

Sample	Langmuir			Freundlich		
	q_max_ (mg/g)	b (L/mg)	RMSE (mg/g)	K_F_ ((mg/g)(L/mg)^(1/n^_F_^)^)	n_F_	RMSE (mg/g)
AV50	346.52	0.018	9.01	4.92	2.83	17.02
AV25	311.81	0.12	10.84	46.81	1.51	41.95
AV0	199.13	0.17	8.74	5.17	2.26	23.48

**Table 2:** Langmuir isotherm parameters for agro-waste adsorbents.

Bio-Adsorbent	T (°C)	pH	$q_{max}\;(\mathrm{mg}/g)$	b	Reference
Papaya seeds	30	7	555.5	0.0028	[25]
Grass waste	30	7	457.6	0.0023	[26]
AV50	20	7	346.52	0.018	[This study]
Rice husk	20	7	312.2	0.0171	[27]
AV25	20	7	311.81	0.12	[This study]
Cotton waste	20	7	277.7	0.009	[27]
AV0	20	7	199.13	0.17	[This study]
Pteris waste roots	20	7	112.3	0.62	[28]
Tea waste	25	8	85.1	1.26	[29]
Peanut hull	20	5	60.05	0.16	[30]
Yerba Mate	25	6	59.6	0.02	[10]
Passion fruit waste	25	8	44.70	0.002	[31]
Apricot shells	25	5	24.31	0.002	[32]
Banana peel	30	7	20.8	0.06	[33]
Chaff	25	7	20.03	0.22	[34]
Spent coffee grounds	25	5	18.72	0.27	[35]
Orange peel	30	7	18.6	0.05	[33]
Wheat shells	30	7	16.56	0.02	[36]

**Table 3:** BET-BJH data for treated *Aloe vera* samples.

Sample	SSA (m^2^/kg)	Mean Pore Diameter (nm)	Total Pore Volume (cm³/kg)
AV0	180.2	9.60	0.11
AV25	313.3	5.11	0.37
AV50	331.7	7.27	0.59

**Table 4:** Langmuir isotherm parameters at 10 °C, 20 °C, and 40 °C for AV0.

Parameter	10 °C	20 °C	40 °C
$q_{max}$ (mg/g)	245.59	199.13	180.21
b (L/mg)	0.18	0.17	0.09

**Table 5:** Kinetic fitting parameters at 20 °C for AV0, AV25, and AV50.

Parameter	AV0	AV25	AV50
$k_{L}a$ (1/min)	0.013 ± 5.30 · 10^−4^	0.014 ± 5.43 · 10^−4^	0.008 ± 3.9 · 10^−4^
$D_{eff,MB}$ (cm^2^/min)	5.43 · 10^−7^ ± 9.02 · 10^−8^	3.89 · 10^−7^ ± 5.92 · 10^−8^	5.78 · 10^−7^ ± 4.30 · 10^−8^
Bi	12.1	13.6	17.9

## Data Availability

Not applicable.

## Supplementary Materials

The following supporting information can be downloaded at: https://www.mdpi.com/article/10.3390/ma15165566/s1, Figure S1: N2 Adsorption Isotherms (for BET analysis); Figure S2: Pore Volume Distribution.
