# Supplementary material for: Effect of Water–Ethanol Extraction as Pre-Treatment on the Adsorption Properties of Aloe vera Waste
Source: Materials (Basel). 2022 Aug 13;15(16):5566. doi: 10.3390/ma15165566 (PMC9412281; doi:10.3390/ma15165566)
Supplement: Supplementary file 1 [file materials-15-05566-s001.zip › materials-1837354-supplementary.pdf]

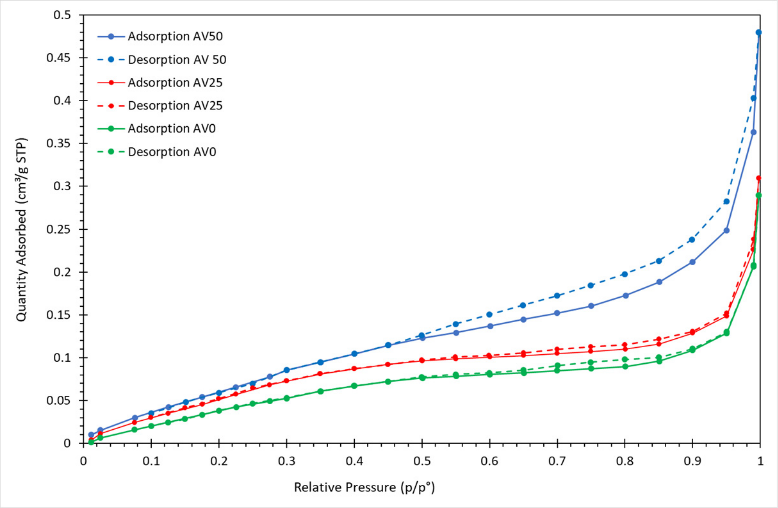

Figure S1. N_2 Adsorption Isotherms(for BET analysis).

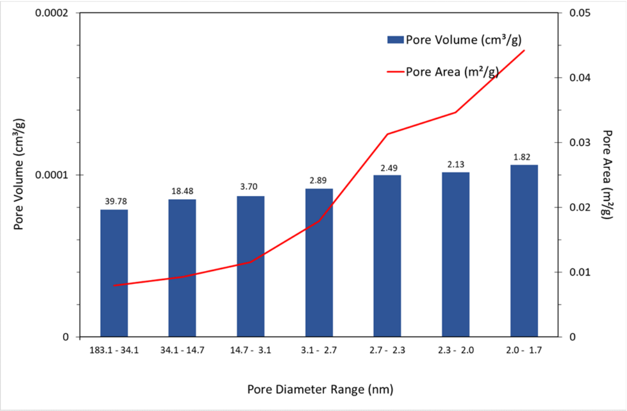

(a) AV0

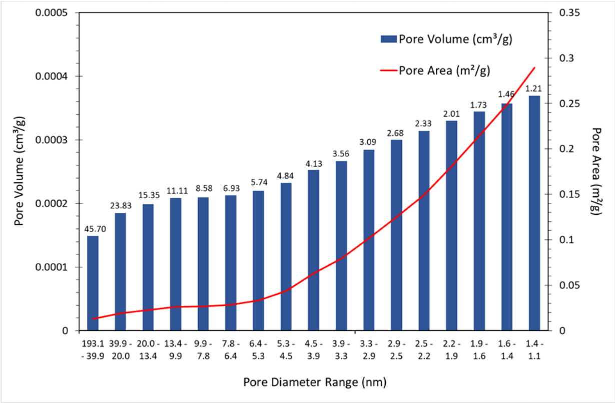

(b) AV25

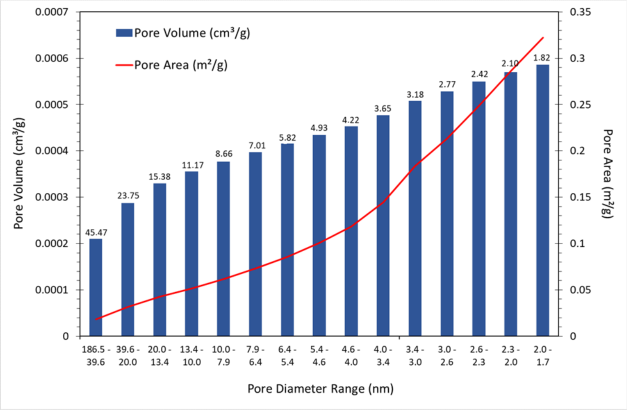

(c) AV50

Figure S2. Pore Volume Distribution.
